# Inference of kinase-signaling networks in human myeloid cell line models by Phosphoproteomics using kinase activity enrichment analysis (KAEA)

**DOI:** 10.1186/s12885-021-08479-z

**Published:** 2021-07-08

**Authors:** Mahmoud Hallal, Sophie Braga-Lagache, Jovana Jankovic, Cedric Simillion, Rémy Bruggmann, Anne-Christine Uldry, Ramanjaneyulu Allam, Manfred Heller, Nicolas Bonadies

**Affiliations:** 1 Department of Hematology and Central Hematology Laboratory, Inselspital, Bern University Hospital, University of Bern, Bern, Switzerland; 2 Department for BioMedical Research (DBMR), University of Bern, Bern, Switzerland; 3 Interfaculty Bioinformatics Unit and Swiss Institute of Bioinformatics, University of Bern, Bern, Switzerland

**Keywords:** Phosphoproteomics, Kinase activity, Kinase-signaling network, Myeloid malignancies

## Abstract

**Background:**

Despite the introduction of targeted therapies, most patients with myeloid malignancies will not be cured and progress. Genomics is useful to elucidate the mutational landscape but remains limited in the prediction of therapeutic outcome and identification of targets for resistance. Dysregulation of phosphorylation-based signaling pathways is a hallmark of cancer, and therefore, kinase-inhibitors are playing an increasingly important role as targeted treatments. Untargeted phosphoproteomics analysis pipelines have been published but show limitations in inferring kinase-activities and identifying potential biomarkers of response and resistance.

**Methods:**

We developed a phosphoproteomics workflow based on titanium dioxide phosphopeptide enrichment with subsequent analysis by liquid chromatography tandem mass spectrometry (LC-MS). We applied a novel *Kinase-Activity Enrichment Analysis* (KAEA) pipeline on differential phosphoproteomics profiles, which is based on the recently published *SetRank* enrichment algorithm  with reduced false positive rates. Kinase activities were inferred by this algorithm using an extensive reference database comprising five experimentally validated kinase-substrate meta-databases complemented with the *NetworKIN* in-silico prediction tool. For the proof of concept, we used human myeloid cell lines (K562, NB4, THP1, OCI-AML3, MOLM13 and MV4–11) with known oncogenic drivers and exposed them to clinically established kinase-inhibitors.

**Results:**

Biologically meaningful over- and under-active kinases were identified by KAEA in the unperturbed human myeloid cell lines (K562, NB4, THP1, OCI-AML3 and MOLM13). To increase the inhibition signal of the driving oncogenic kinases, we exposed the K562 (BCR-ABL1) and MOLM13/MV4–11 (FLT3-ITD) cell lines to either Nilotinib or Midostaurin kinase inhibitors, respectively. We observed correct detection of expected direct (ABL, KIT, SRC) and indirect (MAPK) targets of Nilotinib in K562 as well as indirect (PRKC, MAPK, AKT, RPS6K) targets of Midostaurin in MOLM13/MV4–11, respectively. Moreover, our pipeline was able to characterize unexplored kinase-activities within the corresponding signaling networks.

**Conclusions:**

We developed and validated a novel KAEA pipeline for the analysis of differential phosphoproteomics MS profiling data. We provide translational researchers with an improved instrument to characterize the biological behavior of kinases in response or resistance to targeted treatment. Further investigations are warranted to determine the utility of KAEA to characterize mechanisms of disease progression and treatment failure using primary patient samples.

**Graphical abstract:**

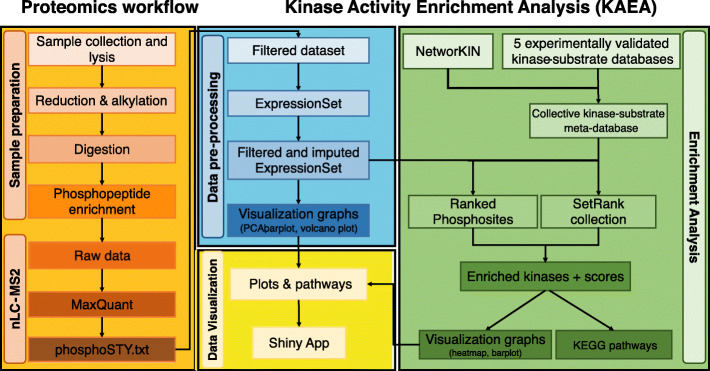

**Supplementary Information:**

The online version contains supplementary material available at 10.1186/s12885-021-08479-z.

## Background

Our understanding of the clonal composition of cancer has substantially advanced in the last decade but we are increasingly facing the limitations of genomics for the prediction of treatment response and the identification of suitable targets of resistance. Initiated by the advent of high-throughput next-generation sequencing (NGS), considerable effort has been devoted to investigate the genomes and transcriptomes of various cancers, including myeloid malignancies [1–3]. These initiatives aimed for a better understanding of individual’s disease biology, identification of prognostic as well as predictive biomarkers and lead to the development of targeted treatments according to the patients’ molecular profiles (precision medicine) [4, 5]. Modern genomics has revolutionized the diagnostic accuracy by its ability to detect previously hidden DNA sequence variations in high-throughput and at single-nucleotide resolution [6]. The evolution of bioinformatics contributed to this development and allowed to reduce the complexity of the data and characterize novel biological clusters [7, 8]. Despite these indisputable achievements of genomics, our understanding of functional biology remains limited.

Conventional cytogenetic analysis was the first genomic approach that was used for diagnostics and disease-based risk stratification [9]. As an example, Chronic myeloid leukemia (CML) was found to be characterized by the BCR-ABL1 translocation and showed later to be a paradigmatic disease amenable to functional cure with tyrosine-kinase inhibitors (TKIs) targeting the ABL1 kinase [10, 11]. Many other TKIs have entered clinics for treatment of hematological malignancies since. These include Ruxolitinib, a janus kinase 1/2 inhibitor for Primary Myelofibrosis and Polycythemia Vera [12], Ibrutinib, a bruton kinase inhibitor for B-cell malignancies [13] and most recently Midostaurin, a multi-kinase inhibitor for FMS-like tyrosine kinase 3 (FLT3) mutated acute myeloid leukemia (AML) [14, 15]. In contrast to CML, most other hematological neoplasms are genetically more heterogeneous and progression under targeted therapies is generally inevitable.

Proteins are biological effectors of the malignant behavior and assumed to reflect more appropriately the functional biology of cancer phenotypes. Phosphorylation is one of the most important post-translational modifications of proteins involved in signal transduction and other important cell functions such as proliferation and energy metabolism [16, 17]. Dysregulation of phosphorylation-based signaling pathways is fundamental for oncogenesis and, therefore, it is not surprising that kinase-inhibitors are attractive targeted therapies in a variety of cancers, including hematological malignancies. Phosphorylation can occur at amino acids serine (86%), threonine (12%) and tyrosine (1.8%) and is conferred by an array of protein kinase families [18]. Phosphoproteomics represents the phosphorylation status of the proteome imposed by kinases and phosphatases at a given time point. There is a growing interest in using kinase inhibitors for the treatment of patients with hematological neoplasms. However, most patients eventually progress and the mechanisms remain frequently obscure. To understand the dynamic roles of the different kinases’ families, multiple groups have attempted to develop different phosphoprotemics approaches to infer the activities of kinases and relate them to the biological state (Table 1). Every pipeline has its unique features but none of these can be currently considered as the optimal “golden standard”.

**Table 1:** Selection of published kinase activity analysis pipelines using phosphoproteomics data

Software	Method	Statistic	Database	Input	Visualization
Kinase-Set Enrichment Analysis (KSEA) [19, 20]	Calculate the ratio of the means of the phosphorylated peptide abundances in the substrate groups relative to their abundances in the whole data set	z-score	PhosphoSitePlus + NetworKIN	Pre-processed comma-separated file with columns of proteins, genes, peptides, phosphosites, *p*-values and Fold changes	KSEA Shiny App
Kinase-Enrichment Analysis (KEA) [21]	Calculate significant deviations from the expected value which is the cardinality of the set of substrates that are targeted by specific kinases divided by the total number of substrates in the background dataset	Fisher’s exact test	NetworKIN + Phospho.ELM + MINT + HPRD + Swiss-Prot + PhosphoPoint +Manual annotations	List of gene symbols	Web tool
Kinase Perturbation Analysis (KinasePA) [22]	In-house directional hypothesis testing framework for pathway analysis	Stouffer’s statistics (z-score)	PhosphoSitePlus +Phospho.ELM	Pre-processed comma-separated file with columns of phosphosites and fold changes	ShinyApp R package
Knowledge-based CLUster Evaluation (CLUE) [23]	Estimate the optimal number of clusters in dataset using K-means based clustering and then identifying the enriched kinases in each cluster	Fisher’s exact test	PhosphoSitePlus	Time-course data set with columns of phosphosites and fold changes at time points	R package
Inference of kinase activities from phosphoproteomics (IKAP) [24]	Non-linear optimization routine to minimize the cost function that relates kinase activities and affinities to phosphosite measurements	–	PhosphoSitePlus	Data set with columns of protein or gene names, the sequences of the measured peptides and data values	Matlab
Integrative Inferred Kinase Activity (INKA) [25]	Inference from single biological samples, combining both kinase- and substrate-centric evidence metrics	INKA score	PhosphoSitePlus + NetworKIN	MaxQuant output files	Web tool
Kinase activity ranking using phosphoproteomics (KARP) [26]	Model the contribution of kinases to cell viability by the net activity of a kinase which is calculated as the sum of intensities of its known substrates relative to the sum of intensities of all phosphorylation sites in the studied dataset.	K-score	PhosphoSitePlus	MaxQuant output files	n.a.

**Fig. 1:**
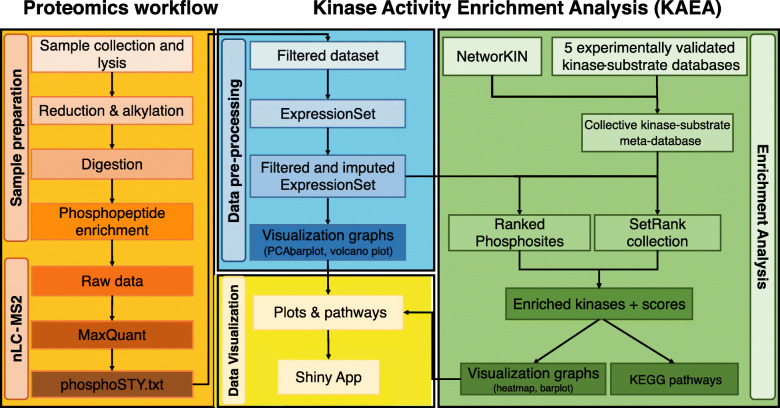
Proteomics workflow and the Kinase-Activity Enrichment Analysis (KAEA) pipeline. For more details see methods section. Manual and source code are publicly accessible on the github repository (https://github.com/Mahmoudhallal/KAEA)

In an attempt to increase the specificity of the enrichment, improve the coverage of kinase-substrate database and provide an interactive visualization of kinases/pathways, we set out in developing a novel untargeted *Kinase-Activity Enrichment Analysis* (KAEA) pipeline (Fig. 1). This pipeline allows inferring kinase- and pathway activities from differential phosphoproteomics mass spectrometry (MS) data by employing a recently published *SetRank* enrichment algorithm, which reduces false positivity rates. Moreover, we use an extensive reference dataset comprising five experimentally-validated kinases-substrates databases that were combined with the *NetworKIN* in-silico prediction tool. Finally, we apply the *ShinyApp* for the interactive visualization of differential phosphosites, enriched kinases, and pathways combined with a subsequent STRING network analysis. Here, we show the development and validation of the KAEA pipeline using human myeloid cell line models exposed to clinically established kinase inhibitors and discuss the distinguishing features compared to other published pipelines.

## Results

### Identification of biologically meaningful kinases in non-perturbed human myeloid cell lines

**Fig. 2:**
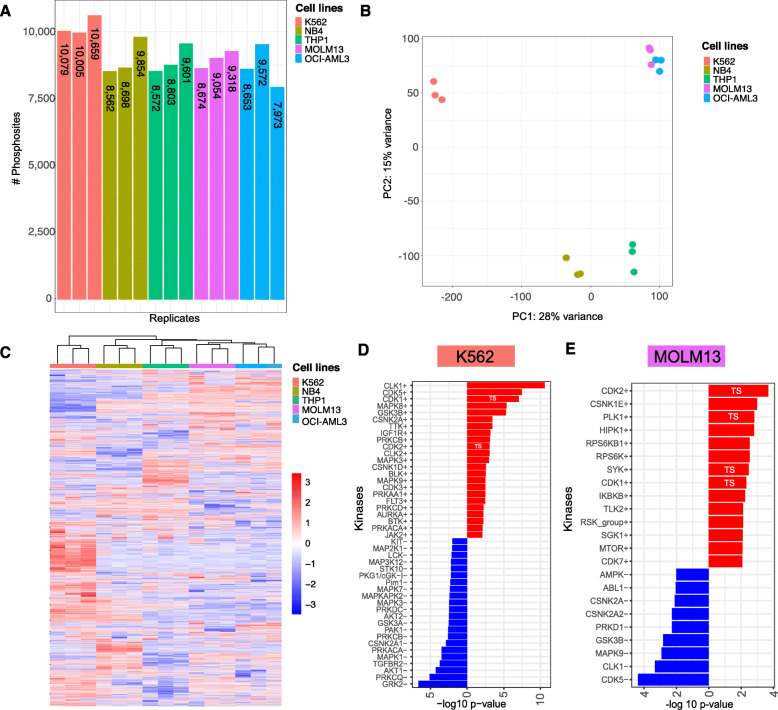
The phosphoproteomes of the unperturbed five human myeloid cell lines. **A** Barplot represents the number of quantified PS in every replicate before imputation for K562 (red), NB4 (olive-green), THP1 (light green), MOLM13 (magenta), and OCI-AML3 (blue). **B** PCA distribution of quantified PS showing phenotypic clusters of cell-lines. **C** Heatmap of row scaled quantified PS showing equivalent clusters as with PCA. **D** KAEA waterfall plot of K562 shows -log10 *p*-values of overactive (red) and underactive kinases (blue) compared to the other four cell lines. **E** KAEA waterfall plot of MOLM13 compared to the other four cell lines. TS: tumor suppressor

In total, 14,590 unique PS were identified and quantified in the pooled replicates of K562, NB4, THP1, MOLM13 and OCI-AML3, respectively (Fig. 2A). Cell lines clustered according to their expected phenotype in erythroid (K562), promyelocytic (NB4), monocytic (THP1) and myelomonocytic (MOLM13 and OCI-AML3) by PCA plot (Fig. 2B) and hierarchical clustering of quantified PS (Fig. 2C).

KAEA was performed on the five cell lines separately, whereby the other four cell lines were used as base line, in order to identify relatively over- and underactive kinases. We focused our analysis on K652 (Fig. 2D) and MOLM13 (Fig. 2E), as these cell-lines are expected to be driven by the oncogenic kinases BCR-ABL1 and FLT3-ITD, respectively. In K562, 23 kinases were overactive and 22 underactive, respectively. ABL was itself not found enriched, but its downstream kinases were, such as Cyclin Dependent Kinases (CDKs), Mitogen-Activated Protein Kinases 3 and 8 (MAPK3/ERK1 and MAPK8/JNK1) [27, 28], Casein Kinase 2A (CSKN2A) [29], and Insulin Like Growth Factor 1 Receptor kinase (IGF1R) [30]. Additional overactive kinases, which have not yet been investigated in detail in the context of BCR-ABL1, included CDC2-Like Kinase 1/2 (CLK1/2), Glycogen Synthase Kinase 3B (GSK3B), Monopolar Spindle 1 kinase (MPS1), also known as TTK Protein Dual Specificity Protein Kinase, and Protein Kinase C Beta (PRKCB).

In MOLM13, 14 kinases were overactive while 9 were underactive. FLT3, by itself, was not found enriched, but its downstream kinases were, such as CDKs, Polo-Like Kinase 1 (PLK1) [31], p90 Ribosomal Protein S6 Kinase (RPS6K, RSK-group) [32, 33], Spleen Tyrosine Kinase (SYK) [34] along with mammalian Target of Rapamycin (mTOR) [35]. Additional overactive kinases, which have not yet been investigated in detail in the context of MOLM13, included Casein Kinase 1E (CSKN1E), Homeodomain Interacting Protein Kinase 1 (HIPK1), Inhibitor of Nuclear Factor Kappa B Kinase Subunit Beta (IKBKB), Tousled Like Kinase 2 (TLK2) and Serum- and Glucocorticoid-Inducible Kinase 1 (SGK1).

Biologically meaningful overactive kinases were also identified in the other three cell lines. This included CSKN2A in PML-RARA driven NB4 cells [36], several kinases involved in the PRK/CREB-signaling in MLL-driven THP1 cells [37, 38] and kinases of the AKT1-pathway in the mutated NPM1-driven OCI-AML1 cells [39] (Suppl. Fig. 1).

Collectively, we found overactive ABL and FLT3 downstream kinases in K562 and MOLM13, respectively, as well as additional meaningful kinases in the other three cell lines as an initial proof of concept for the biological relevance of the generated output of our KAEA pipeline.

### Pharmacological inhibition experiments using specific myeloid cell lines

We reasoned that using the other four cell lines as background control was not ideal, as only relative changes could be enriched interfering with the detection of biologically relevant kinase activities. Therefore, we decided to perform more specific pharmacological kinase inhibition assays using Nilotinib and Midostaurin in the BCR-ABL1 driven K562 as well as FLT3-ITD driven MOLM13/MV4–11 cell lines, respectively.

#### Identification of direct and indirect Nilotinib targets in the BCR-ABL1 driven K562 cell line model

**Fig. 3:**
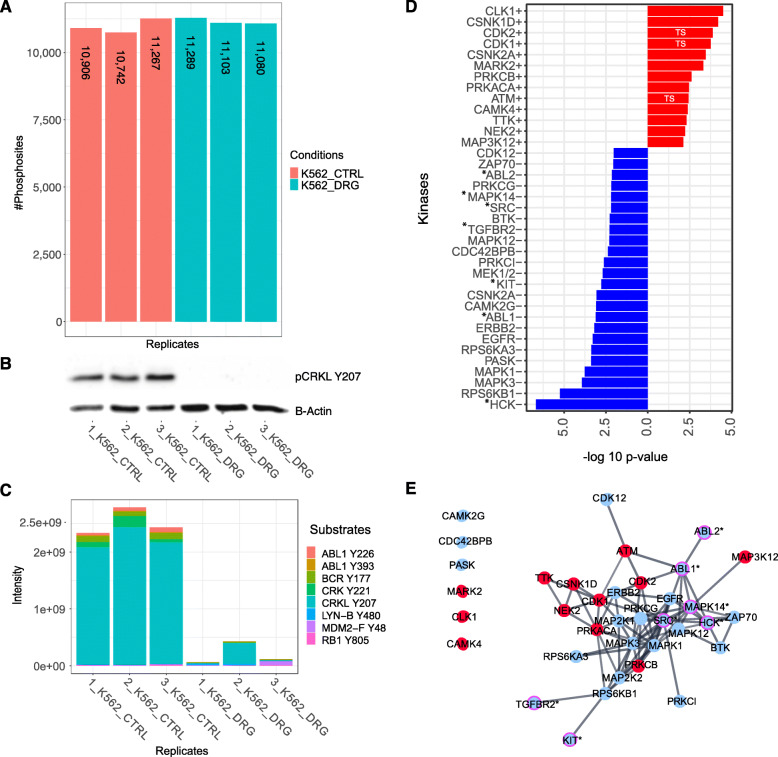
The phosphoproteome analysis of K562 perturbed with Nilotinib. **A** Barplot represents the number of quantified PS in every replicate before imputation for K562 exposed to control (CTRL, red) or 1′000 nM Nilotinib (DRG, green) conditions. (**B**, **C**) Inhibition of ABL1 substrates with most abundant inhibition of the reference site pCRKL Y207 as shown by WB and MS (light green). The original western blots can be found in the additional file 2. **D** KAEA waterfall plot showing overactive (red) and underactive kinases (blue) after exposure of K562 to Nilotinib. **E** STRING kinase-signaling network of significantly positive (red) and negative (blue) enriched kinases. The magenta edged kinases with asterisks (*) highlight experimentally validated targets of Nilotinib. TS: tumor suppressor

We identified in the phosphoproteome of K562 after Nilotinib exposure 4394 protein groups, including 12,617 unique quantified PS (Fig. 3A), of which 7007 and 5610 PS were over- and under-expressed, respectively. Dephosphorylation of pCRKL Y207, as downstream reference site of ABL1 inhibition, was confirmed using western blot (WB) and MS (Fig. 3B, C). KAEA identified 24 inhibited and 13 overactive kinases (Fig. 3D). In contrast, to the experiment performed in the unperturbed cell-lines, here we were mainly interested in the inhibited kinases. Nilotinib inhibition involved the expected direct target kinases Hematopoietic Cell Kinase (HCK), as a member form the SRC proto-oncogenes, ABL1/2, KIT, SRC, MAPK14/p38α [40], and Transforming Growth Factor Beta Receptor 2 kinase (TGFBR2) signaling [41]. In addition, there were other inhibited kinases, which have not yet been investigated in detail in the context of Nilotinib, including RPS6 kinases, MAPK3/ERK1, MAPK1/ERK2, MAPK12/p38γ, PAS Domain-Containing Serine/Threonine-Protein Kinase (PASK), Epidermal Growth Factors Receptor kinases (EGFR, ERBB2), Calcium/Calmodulin Dependent Protein Kinase II Gamma (CAMK2G), Mitogen-Activated Protein Kinase kinases 1/2 (MEK1/2) and PRKCI/PRKCG. Overactive kinases comprised CDKs, CLK1, casein kinases (CSNK1D, CSKN2A), microtubule affinity regulating Kinase 2 (MARK2), PRKCB, CAMK4, as well as the tumor suppressor Ataxia Telangiectasia Mutated (ATM). We characterized the kinase-signaling network with their hubs and interconnected hierarchies using STRING. By this means, we identified MAPK3/ERK1, MAPK1/ERK2, MEK1/2, MAPK12/p38γ, EGFR, PRKCI/PRKCG and ERBB2, as interconnected, down-regulated kinases around the SRC kinase-hub and the tumor suppressors ATM and CDK1/2 as interconnected, up-regulated kinases (Fig. 3E). In summary, our KAEA pipeline identified the expected direct and indirect targets of Nilotinib along with additional, unexplored kinases within a kinase-signaling network in the BCR-ABL driven K562 cell line model.

#### Identification of indirect Midostaurin targets in the FLT3-ITD driven MOLM13 cell line model

**Fig. 4:**
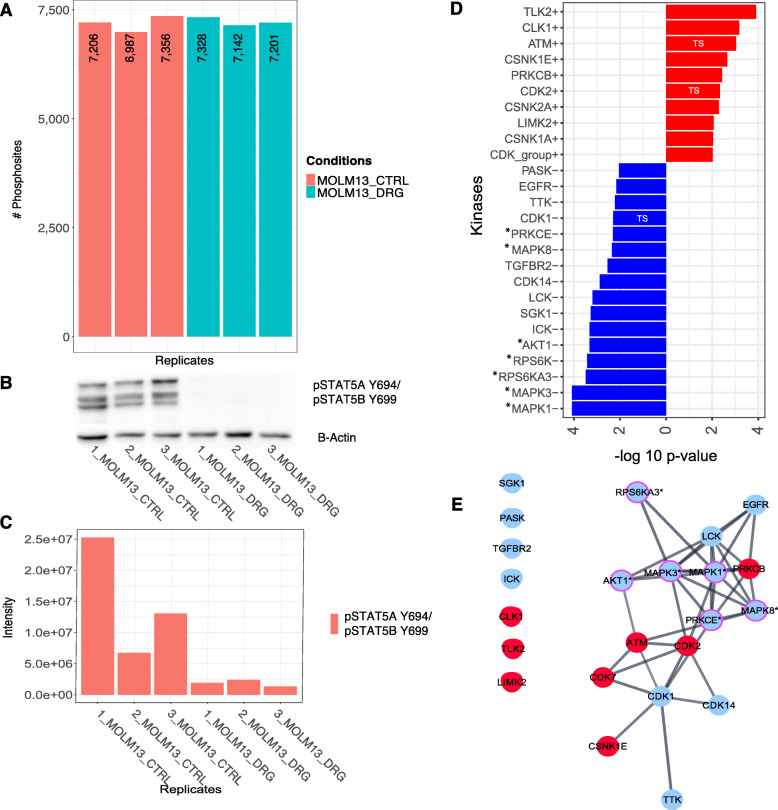
The phosphoproteome analysis of MOLM13 perturbed with Midostaurin. **A** Barplot represents the number of quantified PS in every replicate before imputation for MOLM13 exposed to control (CTRL, red) or 20 nM Midostaurin (DRG, green) conditions. **B**, **C** Inhibition of FLT3 downstream reference site, pSTAT5A/B Y694/Y699 as shown by WB and MS. The original western blots can be found in the additional file 2. **D** KAEA waterfall plot showing overactive (red) and underactive kinases (blue) after exposure of MOLM13 to Midostaurin. **E** STRING kinase-signaling network of significantly positive (red) and negative (blue) enriched kinases. The magenta edged kinases with asterisks (*) highlight experimentally validated targets of Midostaurin. TS: tumor suppressor

We identified in the phosphoproteome of MOLM13 (FLT3-ITD heterozygote) after Midostaurin exposure 3385 protein groups including 8321 unique quantified PS (Fig. 4A), of which 4187 and 4134 were over- and under-expressed, respectively. Dephosphorylation of pSTAT5A/B Y694/Y699, as down-stream reference site for FLT3 inhibition, was confirmed using WB and MS (Fig. 4B, C). KAEA identified 16 inhibited and 10 overactive kinases (Fig. 4D). Midostaurin inhibition of FLT3 was not detected but the expected downstream MAP kinases (MAPK1/ERK2, MAPK3/ERK1, MAK8/JKN1), RPS6K, AKT1 and PRKCE (against which Midostaurin was initially developed) [42]. Additionally inhibited kinases, which have not yet been investigated in detail in the context of Midostaurin, included Intestinal Cell Kinase (ICK), SGK1, Lymphocyte Kinase (LCK), TGFBR2, EGFR and PASK. Overactive kinases comprised CDKs, Tousled-Like Kinase 2 (TLK2), CLK1, Casein Kinases (CSNK1E, CSNK1A, CSNK2A), and the tumor suppressor ATM. We characterized the kinase-signaling network, where we identified LCK and EGFR as interconnected, down-regulated kinases and the tumor suppressors ATM and CDK2 as interconnected, up-regulated kinases (Fig. 4E). In summary, our KAEA analysis identified the expected, but mainly indirect targets of Midostaurin along with additional, unexplored kinases within a kinase-signaling network in the heterozygote FLT3-ITD driven MOLM13 cell line model.

#### Influence of allelic FLT3-ITD burden on Midostaurin kinase inhibition pattern

**Fig. 5:**
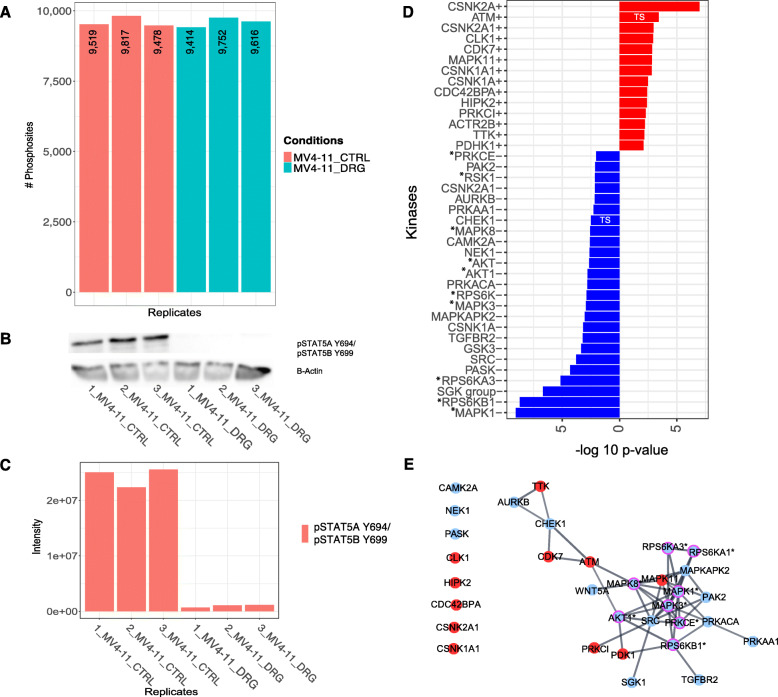
The phosphoproteome analysis of MV4–11 perturbed with Midostaurin. **A** Barplot represents the number of quantified PS in every replicate before imputation for MV4–11 exposed to control (CTRL, red) or 50 nM Midostaurin (DRG, green) conditions. **B**, **C** Inhibition of FLT3 downstream reference, pSTAT5A/B Y694/Y699 as shown by WB and MS. The original western blots can be found in the additional file 2. **D** KAEA waterfall plot showing overactive (red) and underactive kinases (blue) after exposure of MV4–11 to Midostaurin. **E** STRING kinase-signaling network of significantly positive (red) and negative (blue) enriched kinases. The magenta edged kinases with asterisks (*) highlight experimentally validated targets of Midostaurin. TS: tumor suppressor

As we were not able to identify the direct FLT3 inhibition signal in the MOLM13 cell line model, we wanted to investigate, whether a higher FLT3-ITD allelic ratio could potentially increase sensitivity of our analysis pipeline. For this, we used the homozygous FLT3-ITD MV4–11 cell line to compare the kinase inhibition patterns. We identified in the phosphoproteome of MV4–11 after Midostaurin exposure 4309 protein groups including 10,917 unique PS (Fig. 5A), where 5826 and 50941 PS were over- and under-expressed, respectively. Similar to MOLM13, dephosphorylation of pSTAT5A/B Y694/Y699, as downstream reference site for FLT3 inhibition, was confirmed using WB and MS (Fig. 5B, C). KAEA identified 25 inhibited and 14 overactive kinases (Fig. 5D). Again, Midostaurin inhibition of FLT3 was not detected but instead, similarly to MOLM13, the expected, underactive (MAPK1/3/8, AKT/AKT1, RPS6K/RSK1, SGK-group kinases, PRKCE, TGFBR2, and PASK) and overactive (CDK, CLK1, CSNK1A, CSNK2A and the tumor suppressor ATM) kinases. We identified additional, cell-type specific inhibited kinases, which have not yet been investigated in detail in the context of Midostaurin. These included SRC, Glycogen Synthase Kinase (GSK3), MAPK-Activated Protein Kinase 2 (MAPKPK2), NIMA-Related Kinase (NEK1), Protein Kinase CAMP-Activated Catalytic Subunit Alpha (PRKACA), Calcium/Calmodulin-Dependent Protein Kinase Type 2 (CAMK2A), Checkpoint Kinase 1 (CHEK1) and Aurora-Kinase B (AURKB). Additional overactive kinases involved MAPK11, Homeodomain Interacting Protein Kinase 2 (HIPK2) and Pyruvate Dehydrogenase Kinase 1 (PDHK1). We characterized the kinase-signaling network, where we identified SRC, MAPKAPK2, PRKACA, CHEK1 and AURKB as interconnected, down-regulated kinases and the tumor suppressors ATM and CDK7 as interconnected, up-regulated kinases (Fig. 5E). In summary, our KAEA analysis identified similar Midostaurin downstream kinases in the FLT3-ITD hetero- and homozygote cell line models, supporting the robustness of our assay. Additionally, we identified also cell-context dependent kinases within kinase-signaling networks that warrant further investigations.

## Discussion

In our novel KAEA pipeline, we used differential phosphoproteomics profiling data for the untargeted inference of kinase activities in human myeloid cell line models. KAEA allowed the inference of expected direct and indirect kinases inhibited by Nilotinib and Midostaurin, respectively, and the reconstruction of kinase-signaling networks in human myeloid cell line models.

For the initial validation of KAEA, we used five unperturbed human myeloid cell lines with distinct oncogenic driver mutations. In a proof of concept, we focused on K562 and MOLM13, driven by oncogenic kinases ABL1 and FLT3, respectively. As such, we expected higher activities of these two kinases and their interconnected downstream kinases. Intriguingly, we could not detect the direct BCR-ABL1 and FLT3-ITD kinase activities in the unperturbed K562 and MOLM13 cell lines. However, the downstream kinases of ABL1 (MAPK3/ERK1, MAPK8/JNK1, CSKN2A, IGF1R) and FLT3 (PLK1, RPS6K, SYK, mTOR) were significantly enriched in K562 and MOLM13, respectively. We argued that this might be caused by the possibly unsuitable use of the other four cell lines as background, which could have limited the sensitivity to identify cell-type specific differences. To increase the sensitivity of the expected kinase-signals, we decided to perform pharmacological inhibition using clinically established kinase inhibitors.

Nilotinib is a second generation and selective BCR-ABL1 TKI approved for the treatment of CML [43]. It has been reported to target mainly BCR-ABL1 but also other kinases such as KIT and SRC family kinases, which were significantly inhibited in our Nilotinib-K562 perturbation experiment [40]. Our findings were further reinforced by the detection of MAPK1/3 and MEK1/2 inhibition, which are all part of the MAPK pathway downstream of ABL1 and reported to be inhibited also in CML CD34+ cells [44]. The identification of expected, direct and indirect Nilotinib targets validates the ability of our pipeline to detect the biologically relevant kinase activities and the main kinase-signaling network in the BCR-ABL1 driven K562 cell line model.

Midostaurin (PKC412) is a multi-kinase inhibitor approved for the treatment of FLT3-mutant AML [45, 46]. Originally, it was described to inhibit protein kinase C (PKC) and was subsequently found to inhibit also FLT3 kinase [46, 47]. In our experiments, we noticed similar inhibition patterns in the hetero- and homozygote FLT3-ITD cell lines MOLM13 and MV4–11, with inhibition of FLT3 downstream kinases (PRKC, MAPK, AKT, RPS6K). The inhibition of PRKCE underlines the activity of this drug towards PKC family kinases. However, we also found some differences in these two cell line models (SRC, GSK3, CAMK2A, AURKB and RSK1), which underlines the influences of the FLT3-ITD allelic ratio as well as the distinct cell-type context. Intriguingly, in neither of the two perturbation experiments, were we able to detect the direct inhibition of the FLT3 signal. We reasoned that this might be caused by insufficient representation of FLT3 substrates in the MS profile, as only four proteins were represented in our meta-database (FLT3, PDHK1, SHC1, NPM1). This limitation could be potentially mitigated by the inclusion of additional experimentally validated kinase-substrate associations that are involved in the canonical but also oncogenic FLT3 signaling [48, 49]. However, with the dephosphorylation of STAT5 by Midostaurin, we demonstrated unambiguously that the oncogenic FLT3 signaling was inhibited in our model system [50]. The identification of expected, indirect Midostaurin targets validates the ability of our pipeline to detect the biologically relevant kinase activities as well as the main kinase-signaling network in the FLT3-ITD driven MOLM13/MV4–11 cell line models. Interestingly, we found that casein kinases and the tumor suppressor ATM were overexpressed in all cell line models, as potentially shared, cellular and DNA stress reaction during pharmacological exposure [51].

In our pipeline, we used profiling data generated by a MS phopshoproteomics workflow that was developed and established at our proteomics core facility. For our KAEA, we assembled an extensive kinase-substrate meta-database using five published databases that aggregate experimentally observed kinase-substrate associations covering the largest amount of information available from the scientific community. This meta-database was complemented with the *NetworKIN* in-silico kinase-substrate prediction tool, a motif-based kinase substrate inference tool. This meta-database was used as a reference for the *SetRank* enrichment algorithm, which represents the currently largest available evidence as backbone for our KAEA. The *SetRank* algorithm was used due to its stringent behavior as it reduces the false positive rate by avoiding reporting of kinases that are only significantly enriched due to overlapping substrates. It has been previously shown to improve specificity of gene set detection compared to other methods by addressing overlap and multiple testing problems [52]. Currently, there is no optimal “golden-standard” for the inference of kinase activities from phosphoproteomics data available [53] and different approaches have been published (Table 1). A formal comparison between tools, with determination of their accuracy concerning sensitivity and specificity as well as their strengths and limitations, would be desirable but is currently not feasible due to the lack of a reference sample and data standard.

Our KAEA pipeline provided the expected and biologically relevant kinase activities within signaling networks of myeloid cell line models, but we do also acknowledge the potential limitations of our approach. On the level of shotgun MS, insufficient representation of the relevant PS can be a limiting factor and is highly dependent on the quantity, quality and pre-analytical processing of the samples. Data-Independent-Acquisition (DIA) libraries could cover all relevant proteotypes of human myeloid cells and potentially be applied to improve the reproducibility and sensitivity along with reduction of MS running time. Such libraries are not yet available for myeloid malignancies but could be an attractive, standardized reference for the phosphoproteomic profiling of patient’s samples in the future [54]. Another relevant limitation is the incompleteness of our knowledge in substrate-kinase associations. The low representation of PS-kinase associations using all currently published databases was surprising and is a general limitation to the bioinformatics inference of kinase-activities from differential phosphoprofiles. This renders the analysis biased towards the more studied kinases and to those with experimentally observed substrates. The performance will generally improve with the incremental increase in evidence of kinase-substrate associations. Improvements of machine and deep learning algorithms do also affect prediction of kinase-substrate relations. We have used *NetworKIN*, a motif-based in-silico prediction tool, to increase the coverage of kinase-substrates. However, we do also acknowledge that such approaches could add false positive calls as well. Moreover, the STRING network analysis allowed us to identify novel interconnections with known kinases along with unexplored kinases. Focusing on inference of protein kinase activities neglects the relevance of phosphatases, which roles remain generally less clear and substantially under-investigated [55]. A combined protein expression and phosphorylation analysis would uncover both independent and concerted changes in protein expression and phosphorylation [56]. Even if desirable, such approaches are highly resource intensive and hardly implementable in a clinical context, because they require a high amount of proteins, isotope labeling as well as fractionation to generate sufficient overlapping data for protein normalization of dynamic changes in PS. We performed an external validation and analyzed with our pipeline two published datasets using established kinase inhibitors in human cell lines models (PXD001560, PXD004442). Our untargeted approach allowed us to identify the most relevant kinases mentioned in the manuscripts along with components of interacting kinases in other pathways that will need additional investigations (additional file 3). In summary, the analysis of phosphoproteomic profiles has the potential to satisfy an unmet clinical need in characterizing kinase-activities and signaling networks. However, all current pipelines are facing limitations and weaknesses and their outputs are hypothesis generating for thorough biological validation.

## Conclusions

In this study, we developed and validated an improved pipeline for the inference of kinase and pathway activities from differential MS phosphoproteomics profiling data. Our KAEA is based on a recently published enrichment algorithm approach with reduced false-positive rates, an extended reference meta-database of kinase-substrate relations and an interactive visualization tool for differential PS, enriched kinases, and pathways combined with a subsequent STRING network analysis. KAEA allowed the inference of expected and novel kinase activities and their corresponding signaling networks in established human myeloid cell line models. With our pipeline, we provide researchers and clinicians an instrument to monitor biological behavior of kinases in response or resistance to targeted treatment. Further investigations are warranted and ongoing to determine the utility of our pipeline to characterize mechanisms of disease progression and treatment failure.

## Methods

### Cell lines

Human myeloid cell lines K562 (89,121,407, Sigma Aldrich), THP1 (88,081,201, Sigma Aldrich), NB4 (ACC 207, Leibniz Institute DSMZ), MOLM13 (ACC 554, Leibniz Institute DSMZ), OCI-AML3 (ACC 582, Leibniz Institute DSMZ) and MV4–11 (ACC 102, Leibniz Institute DSMZ) were used for our experiments (Suppl. Table 1). The cell lines were cultured in triplicates (quadruplicates for THP1 and OCI-AML1) in suspension using RPMI-1640 (Gibco) supplemented with 10% fetal calf serum (FCS, BioConcept AMIMED) and 1% Penicillin-Streptomycin (15,140,122, Gibco). 20 × 10^6^ cells were harvested for proteomics analysis.

### Antibodies and chemicals

Monoclonal phospho-Stat5a/b Tyr694/Tyr699 (pSTAT5A/B Y694/Y699: 9359 C11C5, Cell Signaling Technology), polyclonal phospho-CrkL Tyr207 (pCRKL Y207: 3181, Cell Signaling Technology), Beta-Actin antibody (sc-47,778, Santa Cruz) were used for western blotting (WB). Nilotinib (AMN107, Sigma Aldrich) and Midostaurin/PKC412 (M1323, Sigma Aldrich) were prepared as 10 mM stock solution in DMSO and stored at − 80 °C.

### Perturbation experiments

Concentration gradient and time course experiments were performed in 48-well plates. WB was used to identify the required conditions for complete de-phosphorylation of the following target proteins: pCRKL Y207 for ABL1 in Nilotinib K562 and of pSTAT5A/B Y694/Y699 for FLT3 in Midostaurin treated MOLM13 or MV4–11. Three replicates of every cell line were cultured for 1 h in control conditions (DMSO) or with Nilotinib in K562 (1000 nM) as well as Midostaurin in MOLM13 (20 nM) or MV4–11 (50 nM), respectively. Higher concentrations for Midostaurin were required for MV4–11 due to their higher FLT3-ITD allelic ratio (FLT3-ITD homozygote).

### Proteomics workflow

#### Cell lysis and protein digestion

Cells were lysed in 8 M urea and 100 mM Tris-HCl pH 8.0 containing complete protease/phosphatase inhibitor cocktail (11,697,498,001, Roche) complemented with 10 mM sodium fluoride and 2 mM di-sodium orthovanadate. Proteins were reduced and alkylated as described elsewhere [57]. Samples were diluted by addition of 1/10-volume of 50 mM Tris HCl pH 8.0 before protein precipitation with 5 volumes of ice-cold acetone over night at − 20 °C. Proteins were pelleted by centrifugation at 16′000 g for 10 min at 4 °C and the acetone supernatant was discarded. Pellets were dried at ambient air for 15 min and stored at − 20 °C until further use.

Proteins were re-dissolved in 8 M urea in 50 mM Tris-HCl pH 8.0 and protein content determined by BCA assay. Urea was then diluted to 1.6 M by addition of 20 mM Tris HCl pH 8.0 with 2 mM calcium dichloride before a two-stage digestion for 2 h at 37 °C followed by over-night at room temperature (RT) with 1/200 (w/w) trypsin-to-substrate ratio each time. Digestions were stopped by adding 1/20-volume of 20% (v/v) tri-fluoroacetic acid (TFA, Fluka) and loading on a pre-conditioned SEP Pak 1 cc C18 cartridge (Waters). The cartridge was washed with 10 volumes of 0.1% TFA before elution of peptides with 1 mL glycolic acid (Sigma Aldrich) at 80 mg/mL in 80% acetonitrile (ACN) / 2.5% TFA (v/v) as loading buffer.

#### Phosphopeptide enrichment

Anatase titanium di-oxide (TiO_2_) beads (Sachtopore NP 5 μm/100 Å, SNX 010S 005) were washed with water and loading buffer before suspending in loading buffer at 100 mg/mL. Phosphopeptides were then enriched with a two-stage extraction procedure, where the first incubation step (INC1) serves as a depletion step for multiply phosphorylated and very acidic peptides, while the second incubation step (INC2) enriches for all remaining phosphopeptides. For INC1, TiO_2_ beads were added to the peptide solution at a ratio of 1:4 TiO_2_/protein (w/w) and incubated under constant shaking for 15 min at room temperature (RT). Beads were spun down and the supernatant transferred into a new vial containing TiO_2_ beads for INC2 at a ratio of 10:1 (w/w) followed by another incubation for 15 min at RT. The last supernatant was diluted to 100 ng/μL with 1% (v/v) TFA and 5 μL subsequently analyzed by nano-liquid chromatography coupled to tandem mass spectrometry (nLC-MS2) as the phospho-depleted compartment.

TiO_2_ beads were then washed several times with 300 μL at constant shaking for 5 min and by discarding all the supernatants with following solutions: i) once with loading buffer, ii) twice with 80 mg/mL glycolic acid in 70% ACN / 0.1% TFA, iii) twice with 70% ACN / 0.1% TFA, iv) and finally twice with 0.1% TFA. Peptides were then eluted twice from TiO_2_ by incubation for 5 min at constant shaking with 50 μL per 1 mg input protein of 50 mM di-sodium hydrogen phosphate / 5 mM sodium orthovanadate / 1 mM sodium fluoride. Both supernatants were transferred into a new vial containing 8 μL formic acid (Merck). INC1 and INC2 eluates were centrifuged for 1 min at 16′000 g and RT before transfer of the supernatant to an HPLC polypropylene vial and 5 μL of both eluates were analyzed separately by MS.

#### Nano-liquid chromatography coupled to tandem mass spectrometry (nLC-MS2)

nLC-MS2 was done by three subsequent injections of each sample on an Orbitrap Fusion Lumos mass spectrometer coupled with a Dionex Ultimate 3000 nano-Ultra Performance Liquid Chromatography (Thermo Fisher Scientific) using a data-dependent acquisition (DDA) method as described elsewhere [58]. nLC-MS2 data interpretation was performed with MaxQuant (version 1.5.4.1) [59] for Trypsin digest. Variable modifications included Oxidation (M), Acetyl (Protein N-term) and Phospho (STY), and fixed modification included Carbamidomethyl (C) only, multiplicity 1, first search at 10 ppm, main search at 4.5 ppm mass accuracy, 0.4 Da mass deviation for the fragment ions. Data was searched against human database (Uniprot Human) with a minimum peptide length of 7 and a false discovery rate (FDR) set at 0.01 for protein, peptides and sites. Incubation 1 (INC1) and incubation 2 (INC2) were analysed together for all experiments except for the five cell lines experiment, which were analysed separately. Our TiO2 based approach is not selective for specific phosphosites (PS) and we found an expected distribution of STY PS in our experiments (mean: S:80.5% ; T:15.8% ; Y:3.75% ) (Suppl. Fig. 2) [18]. The mass spectrometry proteomics source data have been deposited to the *ProteomeXchange Consortium* via the *PRIDE* partner repository (https://www.ebi.ac.uk/pride/) with the dataset identifier PXD024806 [60].

### Kinase activity enrichment analysis (KAEA) pipeline

The complete pipeline is graphically represented in Fig. 1 and consists of three complementary parts: i) data pre-processing, ii) enrichment analysis using a kinase-substrate meta-database and iii) visualization of outputs. The bioinformatics pipeline scripts were written in *R* programming language [61]. *Snakemake* [62], a python-based workflow engine, was used as a wrapper to the *R* scripts to create a reproducible frame for the pipeline. The R based pipeline takes as input *phosphoSTY MaxQuant* output files and returns a list of outputs including qualitative and quantitative evaluations of the dataset alongside the enrichment results. The KAEA manual and source code are publicly accessible on github repository (https://github.com/Mahmoudhallal/KAEA).

#### Data pre-processing

A *YAML Ain’t Markup Language* configuration file defines all the parameters necessary for the down-stream data processing within the pipeline including *MaxQuant phosphoSTY* file path as well as additional parameters (see manual in the repository). The dataset is filtered, normalized, imputed and reorganized to create a *Bioconductor ExpressionSet* class of unique phosphorylation sites (PS). Filtering is done in two steps: 1) potential contaminants and reverse sequences are removed and 2) PS with a localization probability ≥0.75 are kept. Quantiles normalization is applied on the entire log2 transformed dataset.  If the imputation option is set to true, the pipeline sets the missing-at-random data points (conditions where 1 out of 3 technical replicates is a missing value) with Maximum Likelihood Estimate using Expectation-Maximization algorithm and Missing-Not-at-Random data points (conditions where 2 or 3 out of 3 technical replicates are missing values) are imputed by drawing a value from a Gaussian distribution centered around the 0.01th quantile of the sample distribution (MinProb). If the imputation option is set to false, the missing-not-at-random values are set to zero, which gives an enhanced weight to those phosphosites (PS) absent in one condition but present in the other. Results shown here were obtained with the first approach. Rows with only one value in all conditions are not considered in the statistical evaluation.

#### Kinase-substrate meta-database

For our pipeline, we created a meta-database with kinase-substrate associations derived from five publicly available databases 1) *PhosphoSitePlus* (PSP) [63], 2) *Human Protein Reference Database* (HPRD) [64], 3) *Regulatory Network in Protein Phosphorylation* (RegPhos) [65], 4) *The Signaling Network Open Resource* (Signor) [66] and 5) *phospho.ELM* (ELM) [67]. Experimentally observed associations of PS as substrates with kinases were only retained. Kinases with one single entry were removed. In total, our combined meta-database comprises 16,740 unique entries covering 10,045 PS and 426 kinases. Redundancy in kinase names between databases was aggregated manually by using unifying nomenclatures, when possible. The substrate- and kinase-specificities of the meta-database are shown in Suppl. Fig. 3A and B, respectively*.* The motif-based computational prediction tool *NetworKIN* was used, which is based on the network context of kinases and phosphoproteins for the prediction of additional *in-silico* associations [68]. A dataset-specific *NetworKIN* database was produced separately for every experiment based on the PS it encompassed. Experimentally observed kinase-substrate associations from the meta-database are merged with *NetworKIN* predictions specific to every dataset to produce a single combined database. The coverage of quantified PS ranges between 4.6–5.4% with the experimentally observed dataset only and increases to 24.5–26.8% when *NetworKIN* predictions were added.

#### Enrichment analysis

The core part of the enrichment analysis is based on the *SetRank* package (Version 1.1.0) [52]. This enrichment analysis tool was designed to discard gene sets that have been flagged as significant, if their significance is only due to the overlap with another gene set, therefore, eliminating possible false positives. The package has been modified to suit the context of kinase-substrate enrichment. The KAEA requires the previously mentioned meta-database of kinase substrate relations and a ranked list of differential PS. Welsh’s t-test was used to evaluate the different conditions and return a list of PS ranked by their corresponding *p*-values. For over-active and under-active kinases analysis, no cutoff was applied on PS where over- and under-expressed PS were included for both analyses in different ranking. For all our analyses, we used the same threshold of 0.01 p-value and 0.05 FDR. For the five unperturbed cell lines (K562, NB4, THP1, MOLM13, and OCI-AML13), the average of the other four cell lines was used as reference, whereas drug and control conditions were used for the perturbed cell line experiments. *SetRank* uses the ranked list of PS and the kinase-substrate database to perform an enrichment analysis on two levels, 1) enrich for over-active kinase, 2) enrich for under-active kinases. The over- and under-active kinases are shown as -log10 p-values in a waterfall plot in red and blue colour, respectively .

#### Data visualization

An R *ShinyApp* was developed to visualize interactively the differential PS, kinases activities and pathways [69]. The output file of KAEA can be up-loaded in a *ShinyApp*. The application allows the qualitative and quantitative representation of the dataset on the phosphoprotein, phosphopeptide and PS level (histogram, Venn diagram), the differential expression of PS (volcano plot) and the enrichment profile of kinases (barplot, heatmap). Moreover, kinases’ activities are visualized in the context of KEGG pathway diagrams. When available, information on the tumor suppressor (TS) oncogenic context of kinases was included according to the *CancerMine* literature-mined resource [70]. The STRING network analysis was additionally used for the current work within the *Cytoscape StringApp* version 1.5.1 [71–73]. The over- and under-active kinases involved in each cell line were mapped to their STRING defaults, with entries such as MEK1/2 decoupled as MEK1 and MEK2. The STRING overall confidence score gauging the reliability of a protein-protein interaction takes into account evidence from experiments as well as from coexpression analysis, evolutionary signals across genomes, automatic text-mining, and orthology-based transfer of evidence across organisms [73]. A minimum overall confidence score of 0.8 was requested for each edge of the networks shown.

## Supplementary Information

**Additional file 1 **Suppl. Table 1**:** Specifications of human myeloid cell lines. Suppl. Fig. 1**:** The phosphoproteome analysis of the unperturbed five human myeloid cell lines. Suppl. Fig. 2**:** Distribution of Serine (S), Threonine (T) and Tyrosine (Y) PS used in the analysis in the four experiments presented in our manuscript. Suppl. Fig. 3**:** Substrate- and kinase-specificity analysis of meta-database

**Additional file 2.** Western blots

**Additional file 3.** External datasets

## Data Availability

The KAEA manual and source code are publicly accessible on Github repository (https://github.com/Mahmoudhallal/KAEA). Data is available via ProteomeXchange with identifier PXD024806.

## Abbreviations

AML: Acute Myeloid Leukemia
BM: bone marrow
FDR: false discovery rate
KAEA: Kinase Activity Enrichment Analysis
LC-MS: Liquid Chromatography Mass Spectrometry
LP: localization probability
PB: peripheral blood
PS: phosphosites
TiO_2_: Anatase titanium di-oxide
TKI: tyrosine-kinase inhibitor
TS: tumor suppressor
MS: mass spectrometry
NGS: next-generation sequencing
