# Supplementary material for: Inference of kinase-signaling networks in human myeloid cell line models by Phosphoproteomics using kinase activity enrichment analysis (KAEA)
Source: BMC Cancer. 2021 Jul 8;21:789. doi: 10.1186/s12885-021-08479-z (PMC8268341; doi:10.1186/s12885-021-08479-z)
Supplement: Supplementary file 1 — Additional file 1 Suppl. Table 1: Specifications of human myeloid cell lines. Suppl. Fig. 1: The phosphoproteome analysis of the unperturbed five human myeloid cell lines. Suppl. Fig. 2: Distribution of Serine (S), Threonine (T) and Tyrosine (Y) PS used in the analysis in the four experiments presented in our manuscript. Suppl. Fig. 3: Substrate- and kinase-specificity analysis of meta-database [file 12885_2021_8479_MOESM1_ESM.docx]

**Additional file 1:**

**Suppl. Table 1:** Specifications of human myeloid cell lines

| **Cell lines** | **Disease of origin** | **Lineage** | **Driver mutation/ translocation** |
| --- | --- | --- | --- |
| **K562** | Chronic myelogenous leukemia  in blast crisis | Erythroid | BCR-ABL1 |
| **NB-4** | Acute promyelocytic leukemia | Promyelocytic | PML-RARA |
| **THP1** | Acute monocytic leukemia | Monocytic | MLL-MLLT3 |
| **MOLM13** | Acute myeloid leukemia | Myelo-monocytic | Heterozygous  FLT3-ITD |
| **MV4-11** | Biphenotypic B and  myelomonocytic leukemia | Monocytic | Homozygous  FLT3-ITD |
| **OCI-AML3** | Acute myeloid leukemia | Myelo-monocytic | NPM1 gene mutation (type A) |

**Suppl. Figure 1:** The phosphoproteome analysis of the unperturbed five human myeloid cell lines

KAEA waterfall plot of (A) NB4, (B) THP1 and (C) OCI-AML3 showing overactive (red) and underactive kinases (blue) using the four other cell lines as base-line. TS: tumor suppressor.

**Suppl. Figure 2:** Distribution of Serine (S), Threonine (T) and Tyrosine (Y) PS used in the analysis in the four experiments presented in our manuscript.

Our TiO2 based approach is not selective for specific phosphosites (PS) and we found an expected distribution of STY PS in our experiments (S:80.5%; T:15.8%; Y:3.75%) (18).

**Suppl. Figure 3:** Substrate- and kinase-specificity analysis of meta-database

(A) In the substrate-specificity analysis (substrate specificity for kinases), 6,755 PS have only one kinase, 1,910 PS are shared by two kinases, 653 PS by three kinases and the remaining 727 PS by up to 27 different kinases. (B) In the kinase-specificity analysis (number of substrates per kinase), 51 kinases were associated with only two substrates, 34 kinases with three substrates, 26 kinases with four substrates, and the remaining 322 kinases with up to 676 substrates.
