# Supplementary material for: Inference of kinase-signaling networks in human myeloid cell line models by Phosphoproteomics using kinase activity enrichment analysis (KAEA)
Source: BMC Cancer. 2021 Jul 8;21:789. doi: 10.1186/s12885-021-08479-z (PMC8268341; doi:10.1186/s12885-021-08479-z)
Supplement: Supplementary file 2 — Additional file 2. Western blots [file 12885_2021_8479_MOESM2_ESM.pptx]

## Slide 1
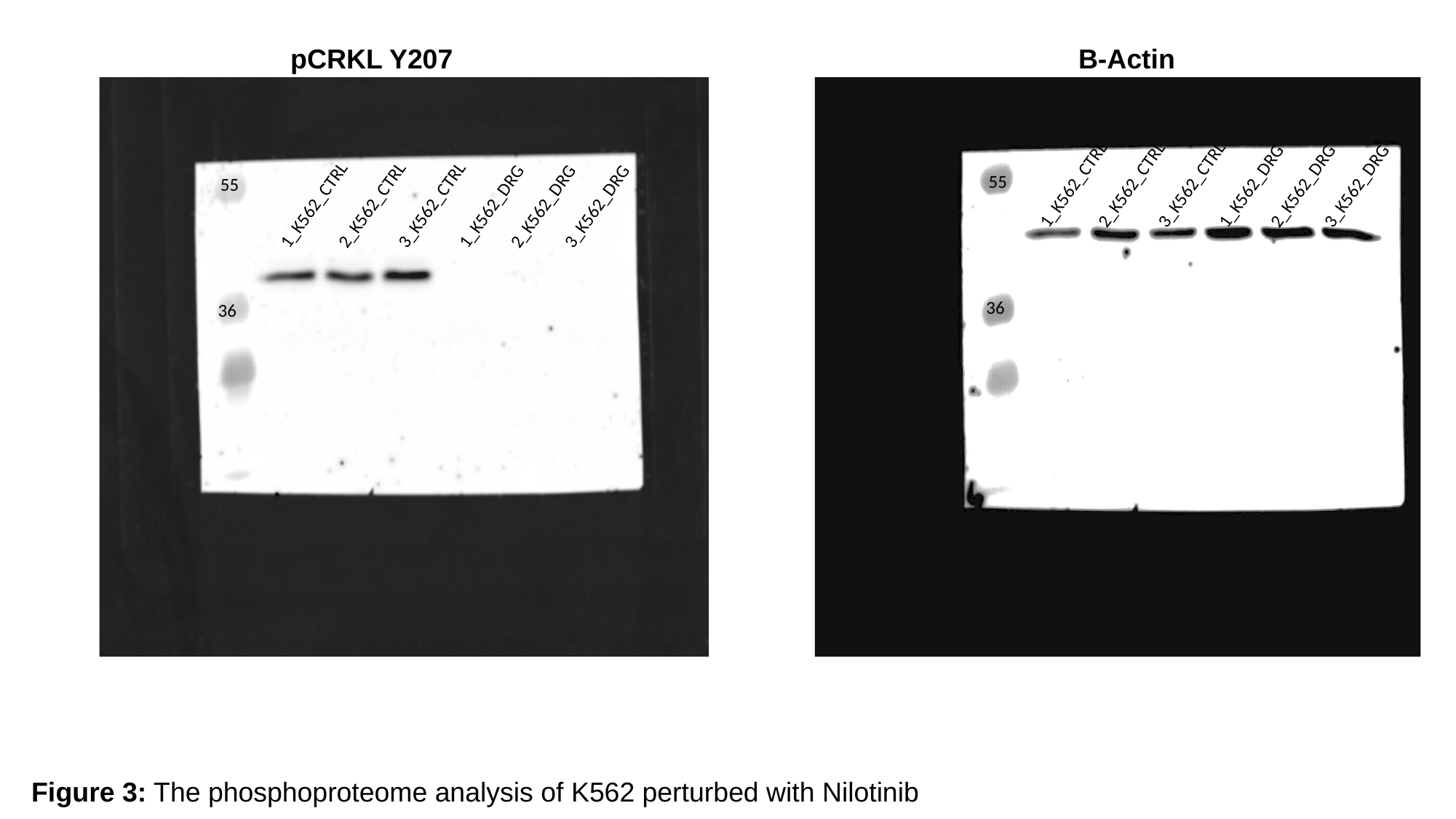

B-Actin
pCRKL Y207
55
1_K562_CTRL
2_K562_CTRL
3_K562_CTRL
55
1_K562_DRG
2_K562_DRG
3_K562_DRG
1_K562_CTRL
2_K562_CTRL
3_K562_CTRL
1_K562_DRG
2_K562_DRG
3_K562_DRG
36
36
Figure 3: The phosphoproteome analysis of K562 perturbed with Nilotinib

## Slide 2
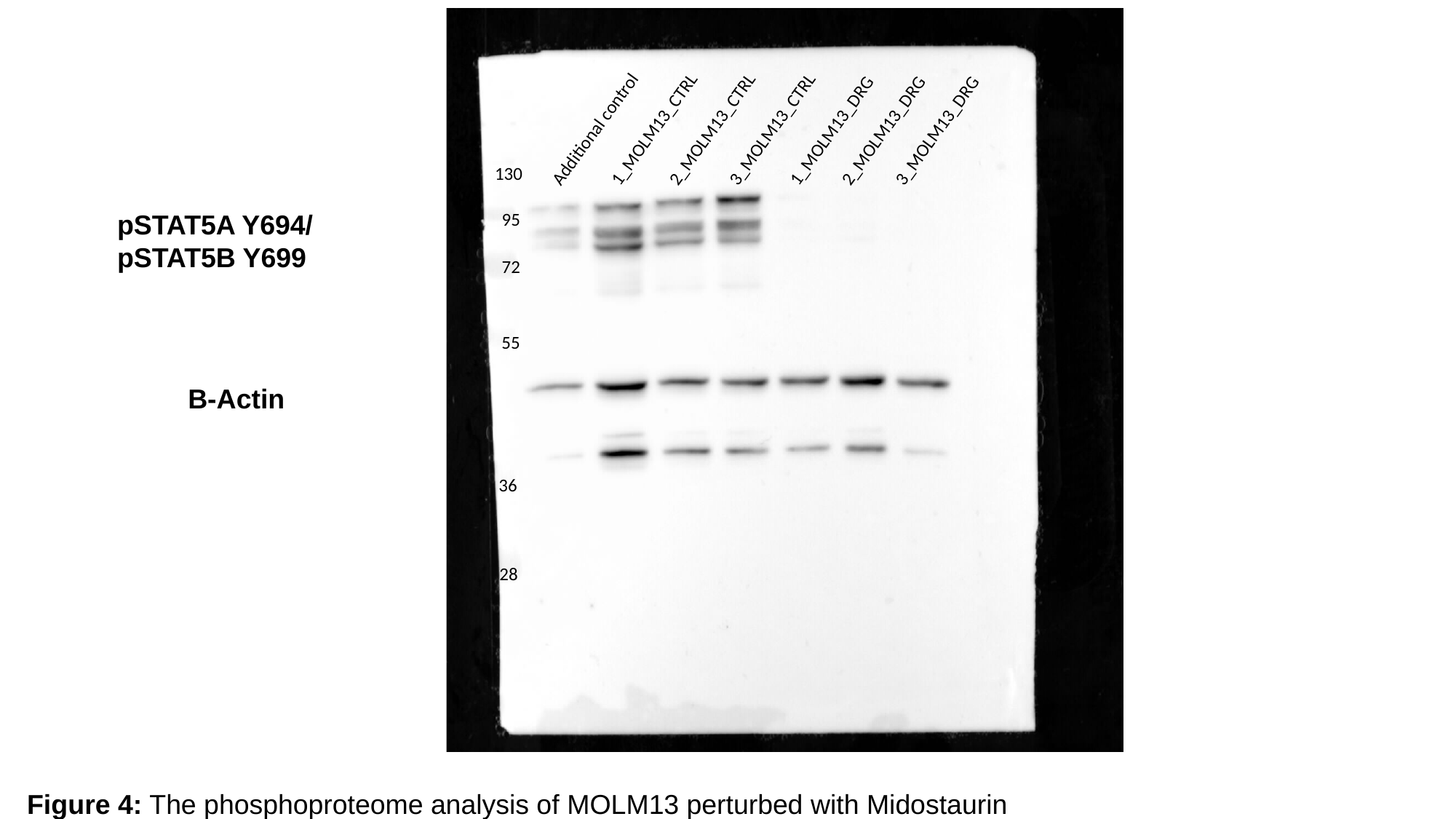

Additional control
1_MOLM13_CTRL
2_MOLM13_CTRL
3_MOLM13_CTRL
1_MOLM13_DRG
2_MOLM13_DRG
3_MOLM13_DRG
130
pSTAT5A Y694/
pSTAT5B Y699
95
72
55
B-Actin
36
28
Figure 4: The phosphoproteome analysis of MOLM13 perturbed with Midostaurin

## Slide 3
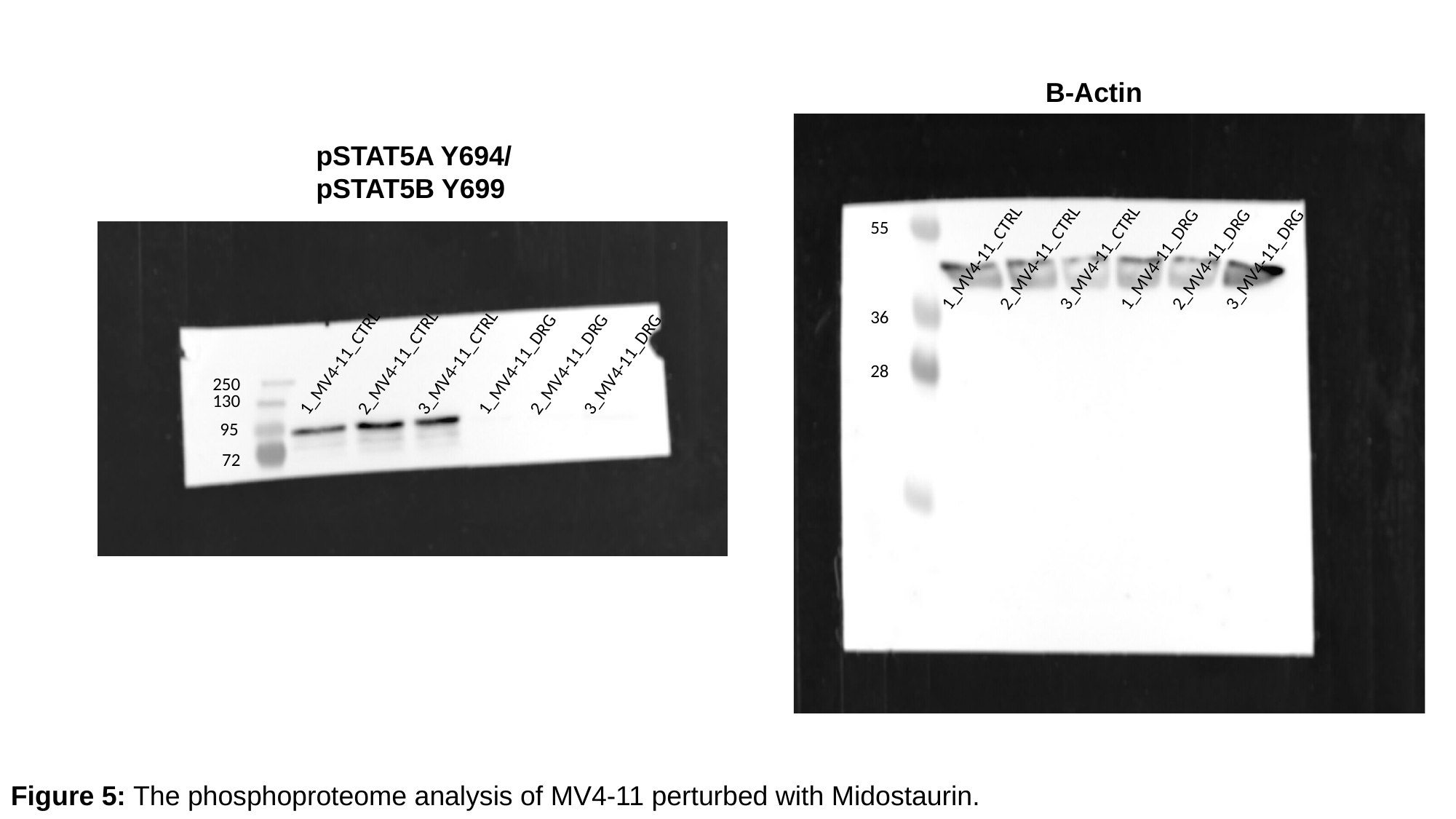

B-Actin
pSTAT5A Y694/
pSTAT5B Y699
55
1_MV4-11_CTRL
2_MV4-11_CTRL
3_MV4-11_CTRL
1_MV4-11_DRG
2_MV4-11_DRG
3_MV4-11_DRG
36
1_MV4-11_CTRL
2_MV4-11_CTRL
3_MV4-11_CTRL
1_MV4-11_DRG
2_MV4-11_DRG
3_MV4-11_DRG
28
250
130
95
72
Figure 5: The phosphoproteome analysis of MV4-11 perturbed with Midostaurin.
