# Supplementary material for: Inference of kinase-signaling networks in human myeloid cell line models by Phosphoproteomics using kinase activity enrichment analysis (KAEA)
Source: BMC Cancer. 2021 Jul 8;21:789. doi: 10.1186/s12885-021-08479-z (PMC8268341; doi:10.1186/s12885-021-08479-z)
Supplement: Supplementary file 3 — Additional file 3. External datasets [file 12885_2021_8479_MOESM3_ESM.docx]

**External dataset 1 (PXD001560): A Phosphoproteomic Comparison of BRAF (V600E) and MKK1/2 Inhibitors in Melanoma Cell Line (1)**

PLX4032 (Vemurafenib) is a BRAF(V600E) inhibitor and AZD6244 (Selumetinib) is a MKK1/2 inhibitor used for the treatment of BRAF(V600E) positive melanoma patients. Here, the authors used these inhibitors to target the RAF/MKK/ERK pathway in a human metastatic melanoma cell line WM239A. Overall, they reported a very high overlap between the phosphosites (PS) in response to AZD and PLX thus the responses to the inhibition of BRAF and MKK1/2 were remarkably uniform. This is clearly visible in our analysis, as shown in figures 2A and 2B, which look almost alike. We could also clearly identify the inhibition of MAPKAPK3, MAPK14, MAP2K1 (MKK1/MEK1), MAPK3 (ERK1), MAPK1 (ERK2) and RPS6KB1 in both experiments pointing towards the inhibition of the expected and reported BRAF/MKK/ERK pathway. Interestingly, they identified 16 PS as responsive only to PLX treatment and 2 PS in response to AZD. Out of these 2 PS responsive only to AZD, one was identified on the ARAF protein. In our analysis, we identified the RAF1 (member of RAF family) as uniquely downregulated in AZD but not in PLX (Figure 2B). On the other hand, they reported that LIMK1 was one of the unique targets of PLX but not of AZD, which we could also find as significantly inhibited in the PLX waterfall plot in our analysis (Figure 2A). Nevertheless, we noticed MAPK1 (ERK2) as slightly overactive in AZD, which can be linked to common PS with other kinases or compensatory mechanisms. The paper’s discussion section indeed highlights that the RIPK2 kinase, which is responsive to PLX but not AZD inhibition, is involved in bypassing MKK1/2 and phosphorylating ERK1/2 directly and could explain our finding. In Figure 2C, we compared the PLX vs AZD to highlight the differential kinase-activity between the two compounds. In conclusion, we have identified the most relevant kinases with our untargeted approach, which goes beyond description of single kinase activates and allows the investigation of interlinked kinase networks.

|  | A    B  C | |  |
| --- | --- | --- | --- |
|  |  |  | |
| **Figure 2:** Dataset PXD001560 analysed with KAEA (0.05 FDR, 0.01 p-value, no imputation) using normalized ratio values without NetworKIN database where (A) WM239A PLX4032 10 µM vs. DMSO, (B) WM239A AZD6244 10 µM vs. DMSO and (C) WM239A PLX4032 10 µM vs. AZD6244 10 µM. | | | |

**External dataset 2 (PXD004442): Phosphoproteome Analysis Reveals Differential Mode of Action of Sorafenib in Wildtype and Mutated FLT3 Acute Myeloid Leukemia (AML) Cell Lines (2)**

Sorafenib is a multikinase inhibitor targeting different receptor tyrosine kinases including FLT3, vascular endothelial growth factor receptor (VEGFR), KIT and RET. In this dataset, they tested sorafenib on the FLT3 positive (MV4-11) and FLT3 negative SKM-1 and MONO-MAC-1 cell lines. The cells were subsequently exposed to DMSO, 0.01 µM sorafenib and 0.2 µM sorafenib. Almost no changes in PS were reported upon exposure to sorafenib in SKM1, which can be clearly observed in our corresponding waterfall plot (Figure 3A), where only a few kinases were enriched as underactive. Nevertheless, one of the few reported upregulated PS in SKM-1 belonged to MEK1 (MAP2K1), which we found as significantly over-active.

MEK1 (MAP2K1), its ERK downstream and RPS6KA1 were shown to be significantly inhibited in MONO-MAC-1 in the published paper. In our analysis, we were able to show inhibition of MAP2K1/2 and RPS6KA1 but surprisingly not for ERKs (Figure 3B). The upstream kinases they identified to be significantly affected in MV4-11 included AKT and RPS6K, and we could confirm AKT1 as the second most inhibited kinase along with RPS6KB1, RPS6KA1, RPS6KA3 and RPS6KA4 in our analysis (Figure 3C). The authors suggested the mTOR pathway to be the most affected in MV4-11. We could indeed enrich for mTOR at 0.01 µM but not at 0.2 µM, which might be related to compensating kinases that emerge at higher concentrations. In the published paper the author concluded that mTOR and MEK/MAPK were the main pathways inhibited in MV4-11 and MONO-MAC-1, respectively, which we were able to identify by our approach. In conclusion, we could again identify the most relevant kinases with our untargeted approach, as well as components of interacting kinases in other pathways that will need additional investigations.

| ****  C  B  A |  |  |
| --- | --- | --- |
| **Figure 3:** Dataset PXD004442 analysed with KAEA (0.05 FDR, 0.01 p-value, no imputation) using normalized ratio values without NetworKIN database where (A) SKM-1 0.2 µM sorafenib vs. DMSO, (B) MONO-MAC-1 0.2 µM sorafenib vs. DMSO and (C) MV4-11 0.2 µM sorafenib vs. DMSO. | | |

Overall, our analyses of two additional datasets using established kinase inhibitors in human cell lines models allowed us to identify expected inhibition of kinase activities as well as candidate interacting kinases and pathways that can be the basis for further experimental validations. **As such, we can hold on to our conclusion that our untargeted approach is suitable for the investigation of kinase-signaling networks.**

**References:**

1. Stuart SA, Houel S, Lee T, Wang N, Old WM, Ahn NG. A Phosphoproteomic Comparison of B-RAFV600E and MKK1/2 Inhibitors in Melanoma Cells. Molecular & cellular proteomics : MCP. 2015;14(6):1599-615.

2. Roolf C, Dybowski N, Sekora A, Mueller S, Knuebel G, Tebbe A, et al. Phosphoproteome Analysis Reveals Differential Mode of Action of Sorafenib in Wildtype and Mutated FLT3 Acute Myeloid Leukemia (AML) Cells. Molecular & cellular proteomics : MCP. 2017;16(7):1365-76.
